# Blood serum amyloid A as potential biomarker of pembrolizumab efficacy for patients affected by advanced non-small cell lung cancer overexpressing PD-L1: results of the exploratory “FoRECATT” study

**DOI:** 10.1007/s00262-020-02788-1

**Published:** 2020-11-24

**Authors:** Vincenzo Di Noia, Ettore D’Argento, Sara Pilotto, Emanuele Vita, Miriam Grazia Ferrara, Paola Damiano, Marta Ribelli, Antonella Cannella, Antonella Virtuoso, Andrea Fattorossi, Giovanni Luca Ceresoli, Michele Milella, Giordano Domenico Beretta, Giampaolo Tortora, Emilio Bria

**Affiliations:** 1 Medical Oncology, Università Cattolica del Sacro Cuore, Rome, Italy; 2 Department of Medical Oncology, Cliniche Humanitas Gavazzeni, Bergamo, Italy; 3 Comprehensive Cancer Center, Fondazione Policlinico Universitario Agostino Gemelli, IRCCS, Rome, Italy; 4 U.O.C. of Oncology, Azienda Ospedaliera Universitaria Integrata, University of Verona, Verona, Italy; 5 Department of Woman and Child Health and Public Health, Fondazione Policlinico Universitario A. Gemelli IRCCS, Rome, Italy

**Keywords:** NSCLC, Pembrolizumab, Serum amyloid A, Biomarker of response, Predictive/prognostic factors to immune-checkpoint inhibitors

## Abstract

**Background:**

Identifying the patients who may benefit the most from immune checkpoints inhibitors remains a great challenge for clinicians. Here we investigate on blood serum amyloid A (SAA) as biomarker of response to upfront pembrolizumab in patients with advanced non-small-cell lung cancer (NSCLC).

**Methods:**

Patients with PD-L1 ≥ 50% receiving upfront pembrolizumab (P cohort) and with PD-L1 0–49% treated with chemotherapy (CT cohort) were evaluated for blood SAA and radiological response at baseline and every 9 weeks. Endpoints were response rate (RR) according to RECIST1.1, progression-free (PFS) and overall survival (OS). The most accurate SAA cut-off to predict response was established with ROC analysis in the P cohort.

**Results:**

In the P Cohort (*n* = 42), the overall RR was 38%. After a median follow-up of 18.5 months (mo), baseline SAA ≤ the ROC-derived cut-off (29.9 mg/L; *n* = 28/42.67%) was significantly associated with higher RR (53.6 versus 7.1%; OR15, 95% CI 1.72–130.7, *p* = 0.009), longer PFS (17.4 versus 2.1 mo; *p* < 0.0001) and OS (not reached versus 7.2mo; *p* < 0.0001) compared with SAA > 29.9 mg/L. In multivariate analysis, low SAA positively affects PFS (*p* = 0.001) and OS (*p* = 0.048) irrespective of ECOG PS, number of metastatic sites and pleural effusion. SAA monitoring (*n* = 40) was also significantly associated with survival endpoints: median PFS 17.4 versus 2.1 mo and median OS not reached versus 7.2 mo when SAA remained low (*n* = 14) and high (*n* = 12), respectively. In the CT Cohort (*n* = 30), RR was not affected by SAA level (*p* > 0.05) while low SAA at baseline (*n* = 17) was associated with better PFS (HR 0.38, 95% CI 0.16–0.90, *p* = 0.006) and OS (HR 0.25, 95% CI 0.09–0.67, *p* < 0.001).

**Conclusion:**

Low SAA predicts good survival outcomes irrespective of treatment for advanced NSCLC patients and higher likelihood of response to upfront pembrolizumab only. The strong prognostic value might be exploited to easily identify patients most likely to benefit from immunotherapy. A further study (FoRECATT-2) is ongoing to confirm results in a larger sample size and to investigate the effect of SAA on immune response in vitro assays.

**Electronic supplementary material:**

The online version of this article (10.1007/s00262-020-02788-1) contains supplementary material, which is available to authorized users.

## Introduction

Immune checkpoint inhibitors (ICIs) are considered one of the most important breakthroughs in non-small cell lung cancer (NSCLC) treatment considering the impressive efficacy and durable activity achievable in a proportion of patients with programmed cell death protein 1 (PD-1) and programmed death-ligand 1 (PD-L1) inhibitors [1].

Over the last 3 years, the anti-PD-1 monoclonal antibodies (mAb) nivolumab and pembrolizumab and the anti-PD-L1 mAb atezolizumab received approval for the treatment of advanced NSCLC after failure of first-line chemotherapy based on the overall survival (OS) benefit over docetaxel demonstrated in phase III trials (CHECKMATE-017 and 057 for nivolumab [2, 3], KEYNOTE-010 [4] for pembrolizumab, OAK [5] for atezolizumab.

The results of KEYNOTE-024 [6] introduced pembrolizumab as the new standard of care in patients with high levels (tumor proportional score, TPS ≥ 50%) of PD-L1 expression. The update survival analysis of this trial continued to demonstrate an OS benefit in favour of pembrolizumab versus standard platinum-based chemotherapy, despite the high crossover rate to pembrolizumab observed in the control group [7]. Moreover, the median OS of 26.3 months (mo) reached by patients with PD-L1 overexpression receiving front-line pembrolizumab represents a new cornerstone in the treatment of NSCLC. Similarly, although in the KEYNOTE-042 trial [8], the OS improvement deriving from pembrolizumab over chemotherapy was demonstrated in the overall cohort with PD-L1 ≥ 1%, the observed benefit was mainly driven by PD-L1 overexpressing cases, which represents in clinical practice only one-third of the overall population of NSCLC patients. Current strategies to extend the benefit of immunotherapy to the majority of patients, including those harbouring PD-L1 low or negative tumors, consist in the combination of PD-(L)1 inhibitors with chemotherapy [9–14] or with other ICIs, such as anti-Cytotoxic T-Lymphocyte Antigen 4 (CTLA-4) mAb [15, 16].

Despite the exciting survival results of pembrolizumab in the KEYNOTE 024 [24], the most of the patients do not respond to single agent immunotherapy as shown by the response rate of 44.8% and it is reasonable that the benefit of pembrolizumab in real-life population appears slightly lower than that observed in the *“more accurately selected trial’s population”*.

Therefore, biomarkers defining patients that are most likely to benefit from ICIs are urgently needed. PD-L1 expression, although its association with better outcomes of pembrolizumab across any line of treatment [17], represents an imperfect biomarker, considering that a strong positivity does not ensure the response with immunotherapy. The high tumour mutation burden (TMB) has been originally reported as a potential predictive biomarker for better outcomes of immunotherapy in NSCLC [18–21]; however, the prognostic rather than predictive role and the lack of measurement standardization limit its exploitation as a routine clinical marker [15, 21]. Recently, the lung immune prognostic index (LIPI) score derived from the combination of baseline neutrophil to lymphocyte ratio (NLR) and lactate dehydrogenase (LDH) was proposed as blood biomarker of efficacy for immunotherapy. However, its role is still debated due to the opposite evidences derived from a multi-institutional retrospective case series [22] and from a further pooled analysis of randomized clinical studies [23].

Clinical data suggest that immunotherapy may be more effective in *“inflamed”* tumor, opening an interesting area of research focused on the exploration of inflammatory components as possible predictive factors for efficacy [24]. Serum amyloid A (SAA) is an acute phase protein with cytokine-like and chemotactic properties, that is markedly up-regulated during various inflammatory conditions [25]. In cancer patients, elevated levels of this protein are not only exclusively connected to the physiological tumour-related inflammation, but also reflect a specific tumour production as potential mechanism of immune evasion, considering the relevant immune-modulating properties of SAA [26].

In this study, we evaluated the association between blood SAA levels and clinical benefit in advanced NSCLC patients during treatment with first-line pembrolizumab, with the final aim to investigate if SAA could represent a potential predictive biomarker for response to anti-PD-1 agents.

## Methods

In this exploratory, observational, prospective, single-center study, blood SAA was collected to be evaluated as a candidate biomarker for first-line pembrolizumab (200 mg iv every 3 weeks) in patients with histologically or cytologically confirmed stage IV NSCLC with PD-L1 TPS of 50% or greater and no sensitizing *EGFR* mutations or *ALK* and *ROS-1* translocations (P cohort). A control cohort of patients with advanced NSCLC and PD-L1 TPS ranges from 0 to 49%, exclusively treated with chemotherapy (CT Cohort), were also prospectively evaluated for SAA. Patients of both cohorts received the treatments at Fondazione Policlinico Universitario A. Gemelli IRCCS.

### Patients’ entry criteria

According to inclusion criteria, patients had undergone no previous systemic therapy for metastatic disease, and had an Eastern Cooperative Oncology Group (ECOG) performance-status (PS) score of 0 or 1 (on a 5-point scale, with 0 indicating no symptoms and higher scores indicating increasing disability). At least one measurable lesion according to Response Evaluation Criteria in Solid Tumors (RECIST) and adequate hematologic, hepatic, and renal functions were required, in addition to normal basal levels of TSH, fT3, fT4 and ACTH for the P cohort. Exclusion criteria for receiving pembrolizumab were symptomatic interstitial lung diseases, autoimmune diseases and systemic immunosuppression. Patients with brain metastases were eligible if asymptomatic or already treated with cranial radiotherapy. Progression was defined according to RECIST 1.1 criteria. CT scan evaluation was performed by a designated radiologist at baseline and every 9 weeks during the treatment. PD-L1 expression was assessed in formalin-fixed tumor samples at local laboratory by a designated pathologist with the use of the commercially available PD-L1 IHC 22C3 pharmDx assay (Dako North America). All adverse events were graded according to the National Cancer Institute Common Terminology Criteria for Adverse Events, version 4.0. Study protocol was reviewed and approved by the local institutional review board and Ethical Committee (Prot. 26,496/19, ID 2640, June 20^th^, 2019) and conducted according to the principles of Declaration of Helsinki and EU General Data Protection Regulation (GDPR, 25.05.18). Patients enrolled in the study signed a written informed consent for biomarkers analysis and clinical data collection at the beginning of therapy. Patients’ data were anonymized before analysis.

### SAA assessment

Blood samples were collected at baseline (day of the first administration of pembrolizumab), before the start of the infusion for both cohorts; the same procedure was performed every 9 weeks in the only P cohort until withdrawal from treatment for either toxicity or disease progression. SAA plasma concentration was measured by a commercial enzyme-linked immunosorbent assay (ELISA) (Siemens Healthineers, Milano, Italy).

### Endpoints and statistical analysis

No power analysis was done to calculate the sample size given the explorative and original aim of the study. The primary end-point response rate (RR) was calculated as the proportion of partial and complete responses assessed by radiological imaging. The secondary end-points were progression-free survival (PFS), defined as the time from randomization until first evidence of objective tumor progression or death from any cause and overall survival (OS), as the time from starting treatment to death for any cause. The endpoints were evaluated according to the SAA value at baseline in both cohorts and during treatment only in the P cohort. SSA was analyzed both as continuous and discontinuous variable (low versus high), using a prefixed threshold value. ROC curve analysis was used to determine the value of SAA levels to adopt as cut-off, which more accurately predict response to pembrolizumab. The association of SAA level with clinico-pathological characteristics and RR was evaluated by Fisher’s exact test, *χ*2 test or Mann–Whitney test, as appropriate, or using Pearson correlation analysis. Estimates of survival times (PFS and OS) were calculated according to the Kaplan–Meier method and compared with log-rank test for the survival univariate analysis. All variables found to be associated (*p* < 0.20) with PFS, OS and RR in the univariate model were included in the multivariate analysis, using the Cox proportional-hazards regression model. Each analysis was performed with the use of a two-sided 5% significance level and a 95% CI. Data were analyzed using MedCalc (v.18.5).

## Results

### Pembrolizumab cohort

#### Patients’ characteristics and overall efficacy and safety data

From July 2017 to December 2018, 42 consecutive patients were enrolled. Characteristics of patients are reported in Table 1. Median age was 70.5 years (range 35–86); 30 patients (71%) were males, 37 (88%) were current or former smokers, ECOG PS was 1–2 for 26 (62%) patients. Histology was non-squamous in 36 (86%) and squamous in 6 (14%) patients. The number of metastatic sites were ≥ 3 in 24 (57%) patients. The overall RR was 38% (95% CI 25–53) and DCR 60% (95% CI 45–75), with 16 responses (14 partial and 2 complete) and 9 stable disease achieved. There were no significant correlations between RR and age, sex, ECOG PS, smoking history, histology, Body Mass Index (BMI) categories, comorbidities (including ischemic heart disease, diabetes mellitus, chronic obstructive bronchitis), number of metastatic sites, brain metastases, liver metastases, pleural effusion, prior thoracic radiotherapy, immune-related toxicity, prior steroid use (Table 1S).

**Table 1:** Baseline characteristics of the patients in P cohort

	No. patients (42)	% 100
Age (years)		
Median (range)	70.5 (35–86)	–
≥ 65	27	64
< 65	15	36
Sex		
Male	30	71
Female	12	29
ECOG performance status		
0	16	38
1	24	57
2	2	5
Smoking history		
Current or former smokers	37	88
Never smokers	5	22
Hystology		
Squamous	6	14
Non-squamous	36	86
No. of metastatic sites		
1	2	5
2	16	38
3	17	41
≥ 4	7	16
Brain metastases		
No	27	64
Yes	15	36
Site of mestastases		
Pleural effusion	13	31
Liver	3	7
Lung	25	59
Bone	12	29
Mediastinal Nodes	34	80
Adrenal Glands	10	23
Other	8	19
BMI categories		
Underweight < 18.5	2	5
Normal weight 18.5–24.9	28	67
Overweight or obesity ≥ 25	12	28
Comorbidities		
No	20	47
Yes	22	53
Prior thoracic radiotherapy		
No	18	43
Yes	24	57
Steroid use		
No	28	67
Yes	14	33

*No* number

After a median (m) follow-up time of 18.5 mo, the mPFS of overall population was 8 (95% CI 4.4–17.4) mo. The mOS was not reached; at 6 months, 92.5% of patients were alive. Immune-related adverse events of any grades were observed in 19/42 (45%) patients. The most frequent were grade 2 pneumonitis (8, 19%), grade 1 thyroid function disorders (5, 12%), grade 1 asthenia (3, 7%). All pneumonitis resolved after short-term treatment discontinuation and high-dose steroid therapy. One case of grade 3 hepatitis occurred concurrently with disease progression, thus the treatment was stopped and steroid were administrated until resolution.

#### Baseline SAA analysis

**Fig. 1:**
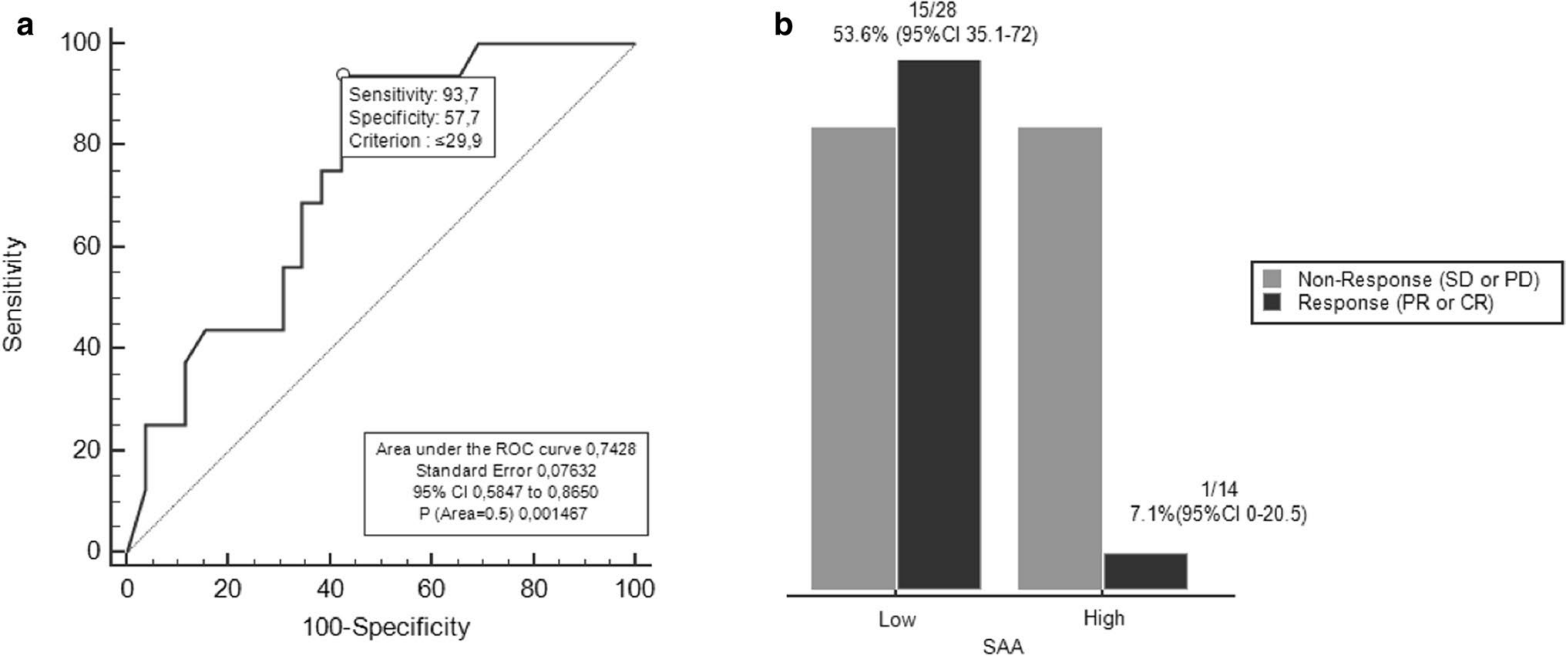
**a** ROC curve for baseline SAA and RR in P cohort. **b** Baseline SAA and RR in P cohort

Median SAA level at baseline in all patients was 16.8 mg/L (2.2–718 mg /L). Using the ROC curve to determine the best value of baseline SAA to predict response, we identified ≤ 29.9 mg/L (AUC 0.74, 95% CI 0.59–0.87, *p* = 0.002) as the cut-off point that combined maximal sensitivity (93.7%, 95% CI 69.8–99.8%) with best specificity (57.7%, 95% CI 36.9–76.6%) (Fig. 1a). Adopting the ROC-derived threshold, 28 (67%) patients were classified as having “low” SAA, while 14 (33%) were categorized as having SAA “high”. The association between clinical characteristics and SAA level groups are reported in Table 2. Only metastatic sites ≤ 2 was significantly correlated with low SAA level (*p* = 0.010). SAA concentrations was not associated with neither BMI of patients (*r* = 0.16, *p* = 0.300) and PD-L1 expression (*r* = 0.15, *p* = 0.330).

**Table 2:** Relationship between baseline SAA and clinical characteristics in P Cohort

	*p* value	Low SAA		High SAA	
		no	%	*n*	%
Age (in years)	0.515				
< 65		9	60	6	40
≥ 65		19	70	8	30
Sex	0.718				
Male		19	63	11	37
Female		9	75	3	25
ECOG PS	0.179				
0		13	81	3	19
1–2		15	58	11	42
Smoking history	0.649				
Never smokers		4	80	1	20
Current or Ex smokers		24	65	13	35
Hystology (Sq vs Non sq)	1.000				
Squamous		4	67	2	33
Non-squamous		24	67	12	33
BMI categories (kg/m^2^)	0.634				
< 18.5		1	50	1	50
18.5–24.9		20	72	8	28
≥ 25		7	58	5	42
Comorbidities (No vs yes)	0.108				
No		16	80	4	20
Yes		12	55	10	45
No. of metastatic sites	**0.010**				
1–2		16	89	2	11
≥ 3		12	50	12	50
Brain metastases (No vs Yes)	0.193				
No		20	74	7	26
Yes		8	53	7	47
Liver metastases (No vs Yes)	0.253				
No		27	69	12	31
Yes		1	33	2	67
Pleural effusion (No vs Yes)	0.082				
No		22	76	7	24
Yes		6	46	7	54
Prior thoracic radiotherapy	1.000				
No		16	67	8	33
Yes		12	67	6	33
IrAE (No vs yes)	1.000				
No		15	65	8	35
Yes		13	69	6	31
Steroid use	1.000				
No		19	68	9	32
Yes		9	64	5	36

*BMI* Body Mass Index, *IrAE* Immune-related Adverse Events, *no* number

#### Response rates and baseline SAA

RR was significantly higher in patients with low SAA compared with SAA high group (53.6 versus 7.1%, OR 15, 95% CI 1.72–130.7, *p* = 0.009) (Fig. 1b). In responding patients, (*n* = 16) median SAA levels at baseline were significantly lower (9.9 mg/L, 95% CI 4.7–18.6) than in non-responders (*n* = 26) (55.9 mg/L, 95% CI 10.2–106) (*p* = 0.009). At multivariate analysis, low SAA was confirmed as an independent predictor for treatment response (OR 14.6, 95% CI 1.49–142.68, *p* = 0.003).

#### Survival analysis and baseline SAA

**Fig. 2:**
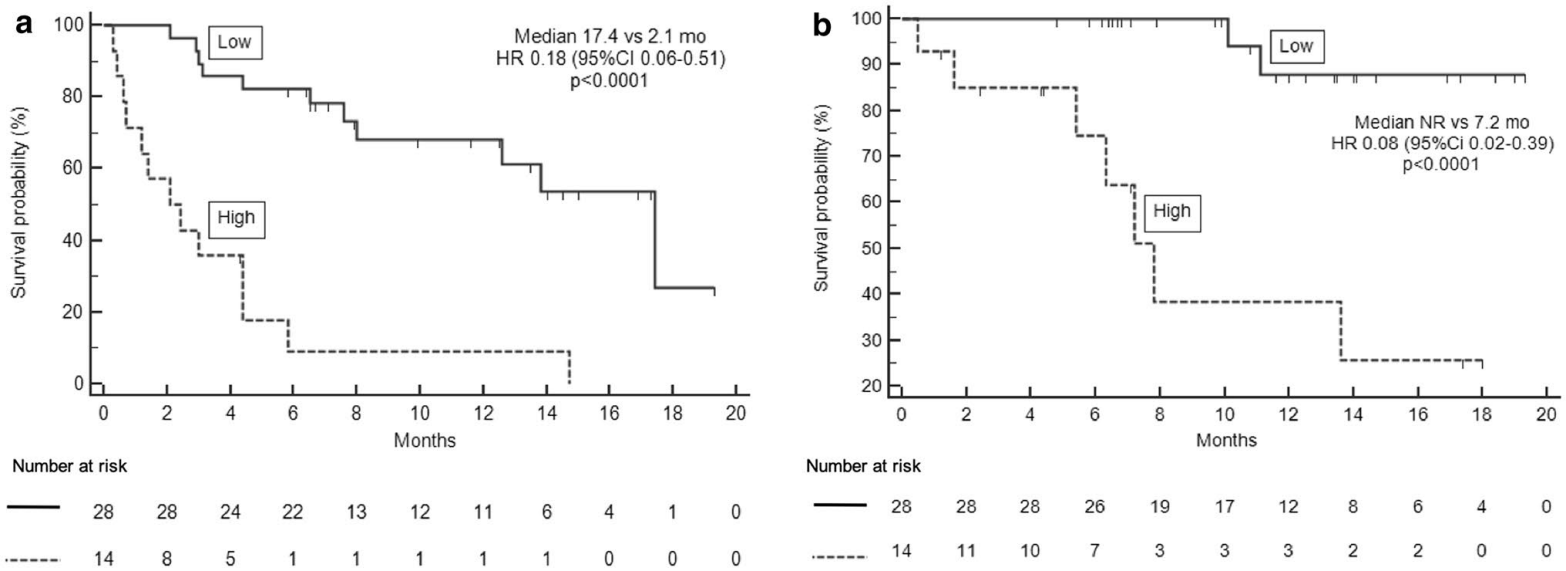
PFS** a** and OS** b** according to baseline SAA in P cohort

Patients having “low” SAA reached longer PFS (17.4 versus 2.1 mo, HR 0.18, 95% CI 0.06–0.51, *p* < 0.0001) than those with “high” SAA (Fig. 2a). At univariate analysis, also ECOG PS 0, metastatic sites ≤ 2 and absence of pleural effusion before treatment initiation were significantly associated with better PFS (Table 3a). Multivariate analysis confirmed “low” SAA (*p* = 0.001) and the absence of pleural effusion (*p* = 0.023) before treatment initiation as independent predictors for longer PFS. Patients with “low” SAA had longer OS in comparison with patients belonging to “high” SAA group (not reached [NR] versus 7.2 mo, HR 0.08, 95% CI 0.02–0.39, *p* < 0.0001) (Fig. 2b). Absence of pleural effusion before the first cycle was also significantly associated with longer OS (HR 0.19, 95% CI 0.05–0.85, *p* = 0.010). Multivariate analysis for OS showed that only “low” SAA independently predicted a good prognosis (HR 0.17, 95% CI 0.03–0.98, *p* = 0.048) (Table 3b).

**Table 3:** (a) PFS according to baseline SAA and clinical characteristics of patients in P Cohort. (b) OS according to clinical and molecular characteristics of patients in P Cohort

	Median PFS (mo)	Univariate	*p*	HR	Multivariate	*p*
		PFS HR (95% CI)			95% CI	
(a)						
Baseline SAA (Low vs high)	17.4 vs 2.1	0.17 (0.06–0.51)	** < 0.0001**	0.13	0.02–0.71	**0.019**
Age (< 65 vs ≥ 65 years)	7.6 vs 13.8	1.82 (0.77–4.31)	0.127			
Sex (Male vs Female)	8 vs 6.5	0.78 (0.30–1.98)	0.579			
ECOG PS (0–1 vs 2)	17.4 vs 6.5	0.43 (0.11–1.64)	**0.001**	NS	NS	NS
Smoking history (Never vs Current or Ex)	4.4 vs 12.6	1.39 (0.41–4.69)	0.532			
Hystology (Sq vs Non sq)	7.6 vs 12.6	1.47 (0.48–4.51)	0.419			
BMI Categories (< 25 vs ≥ 25 kg/m2)	13.8 vs 4.4	0.55 (0.21–1.39)	0.141	NS	NS	NS
Comorbidities (No vs Yes)	8 vs 5.8	0.96 (0.43–2.14)	0.917			
No. of metastatic sites (< 3 vs ≥ 3)	17.4 vs 4.4	0.41 (1.18–0.91)	**0.033**	NS	NS	NS
Brain metastases (No vs Yes)	14.1 vs 11.1	0.76 (0.31–1.86)	0.503			
Liver metastases (No vs Yes)	8 vs 14.7	1.22 (0.32–4.64)	0.779			
Pleural effusion (No vs Yes)	17.4 vs 4.4	0.28 (0.11–0.74)	**0.001**	3.54	1.19–10.52	**0.023**
Prior thoracic radiotherapy (No vs Yes)	12.6 vs 7.6	0.90 (0.39–2.09)	0.811			
IrAE (No vs Yes)	14.7 vs 5.8	0.82 (0.29–1.45)	0.280			
Steroid use (No vs Yes)	5.8 vs 8	0.91 (0.38–2.17)	0.843			

	HR	Univariate	*p*		Multivariate	
		OS HR (95% CI)				
(b)						
Baseline SAA (Low vs high)		0.08 (0.02–0.39)	0.0001	0.17	0.03–0.98	**0.048**
Age (< 65 vs ≥ 65 years)		0.53 (0.13–2.01)	0.336			
Sex (Male vs female)		0.36 (0.08–1.64)	0.325			
ECOG PS (0–1 vs 2)		0.38 (0.10–1.42)	0.208			
Smoking history (Never vs Current or Ex)		0.58 (0.10–3.21)	0.588			
Hystology (Sq vs non sq)		0.87 (0.12–6.25)	0.896			
BMI Categories (< 25 vs ≥ 25 kg/m2)		0.71 (0.16–3.16)	0.636			
Comorbidities (No vs yes)		0.74 (0.20–2.74)	0.653			
No. of Metastatic Sites (< 3 vs ≥ 3)		0.20 (0.05–0.77)	0.088		NS	
Brain Metastases (No vs yes)		0.28 (0.07–1.09)	0.054		NS	
Liver Metastases (No vs yes)	9 vs 7 mo	0.86 (0.44–1.66)	0.624			
Pleural effusion (No vs yes)		0.19 (0.04–0.84)	**0.010**		NS	
Prior thoracic Radioterapy (No vs yes)		0.71 (0.18–2.77)	0.607			
IrAE (No vs yes)		0.82 (0.22–3.05)	0.777			
Steroid Use (No vs yes)		0.49 (80.12–1.88)	0.278			

*CI* confidence interval, *HR* hazard ratio, *IrAE* Immune-related Adverse Events, *OS* overall survival

#### SAA monitoring during treatment

**Fig. 3:**
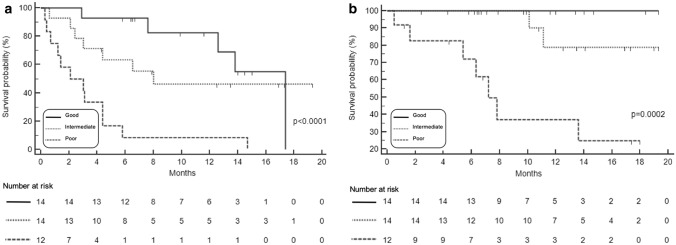
PFS **a** and OS **b** according to SAA at baseline and during the treatment in P cohort

Considering SAA at baseline and during pembrolizumab course, the 40 evaluable patients were classified into 3 groups. The “good profile” group included 14 (35%) patients having “low” SAA at baseline and during treatment. The “poor profile” group included 12 (30%) patients with “high” SAA at baseline and under treatment. At “intermediate profile” group belong 14 (35%) patients who changed SAA level during treatment from “high” to “low” and vice versa. “Good profile” patients had longer PFS than patients belonging to “poor profile” group (17.4 versus 2.1 mo, HR 0.16, 95% CI 0.05–0.48, *p* < 0.0001). The median PFS of 8 mo (95% CI 3–8) reached by patients classified as “intermediate profile” was significantly longer in comparison with “poor profile” patients (HR 0.26, 95% CI 0.08–0.84, *p* = 0.0002) (Fig. 3a). The “good profile” classification was significantly associated with longer OS compared with the other groups (*p* = 0.0002). The median OS was not reached at 18-mo in “good” and “intermediate” profile groups while was 7.2 mo (95% CI 5.6–13.4) in “poor” profile group. No deaths were recorded in “good” profile group at current data lock (Fig. 3b).

### Chemotherapy cohort

#### Patients’ characteristics, overall efficacy and baseline SAA analysis

In the chemotherapy cohort, from July 2018 to December 2019, 30 patients were included. Baseline characteristics are summarized in Table 2S and were comparable with clinical features of P cohort, except for PD-L1 expression. All patients received a platinum-based doublets chemotherapy.

After a median follow-up of 18.7 mo, mPFS and mOS were 4.3 (95% CI 2.7–6.4) and 11.4 (95% CI 7.7–13.3) mo, respectively. Median SAA at baseline was 15.1 mg/L (range, 3.30–597.00).

According to the fixed cut-off, 17 patients (56%) had a “low” SAA and 13 (44%) “high” SAA. No correlation was observed in the CT cohort between baseline SAA and RR (Fig. 1S). “Low” SAA at baseline was associated with better PFS (6.4 versus 2.7 mo, HR 0.38, 95% CI 0.16–0.90, *p* = 0.006) and OS (15.4 versus 7.7 mo, HR 0.25, 95% CI 0.09–0.67, *p* = 0.0002) compared with pre-treatment “high” SAA (Fig. 2S).

### Discussion

In the present prospective study, we found that NSCLC patients with “low” SAA at baseline had a greater likelihood to respond to first-line pembrolizumab than those with higher pre-treatment SAA, while responses to chemotherapy was not affect by SAA level. Moreover, patients having “low” SAA reached longer PFS and OS irrespective of treatment received (pembrolizumab or chemotherapy) than those harboring “high” SAA.

We hypothesized that blood level of tumor-derived SAA could be useful to predict response to anti-PD-1 mABs in NSCLC patients, basing on the immune-modulating properties of SAA and its possible involvement in the process of tumor escape from immune-surveillance.

The acute-phase SAA proteins exhibit significant pro-inflammatory activity, by inducing the synthesis of several cytokines and promoting chemotaxis for monocytes and neutrophils, and act as apolipoprotein of high-density lipoprotein (HDL), involved in the reverse cholesterol transportation during inflammatory states [25]. SAA was also involved in the pathogenesis of the “secondary” amyloidosis and others chronic inflammatory diseases, such as rheumatoid arthritis, atherosclerosis, Alzheimer’s disease and cancer.

Elevated levels of SAA in blood were detected relatively early in cancer patients and, thus, several studies have evaluated SAA as possible serum biomarker for various subtype of cancer, including ovarian, lung, renal, endometrial, uterine and melanoma [26], without drawing any definitive conclusion. Afterwards, the correlation between SAA and cancer was reinvigorated by the finding of local SAA production within tumor tissues, which was significantly higher in lung cancer patients than in other pulmonary disease or other cancer types [27]. In melanoma patients, tumor-derived SAA regulated the plasticity of polymorphonuclear myeloid-derived suppressor cells (PMN-MDSCs) through the binding to the Formyl peptide receptor 2 (FPR-2) on the surface of these cells, resulting in the expansion of IL-10-secreting PMN-MDSCs, which downregulated T-cell anti-tumor activity [28]. At the same time, as negative feedback mechanism, SAA stimulated iNKT (invariant Natural Killer T) cells to interact with IL-10-secreting PMN-MDSCs, a process that inhibits their immune-suppressive activity by reducing IL-10 and enhancing IL-12 production. It is conceivable that tumors produce SAA as immune-escape mechanism, such as the PD-L1 expression, exploiting the capability of SAA to impair the function of T-cells, which represent the main players in anti-cancer immune-response elicited by PD-(L)-1 inhibitors.

The survival findings of our study suggest that SAA may have a prognostic rather than predictive significance when evaluated as biomarker for immunotherapy. However, given the immune-suppressive property on antitumor response described above and the observed strong correlation with response to pembrolizumab and not to chemotherapy, a potential effect of SAA on the activity of immunotherapy cannot be excluded. Remarkably, response rate was adopted as primary endpoint for defining the SAA cut-off to exploit its direct correlation with drug activity with less risk of confounding factors than survival endpoints.

However, this strong prognostic value of SAA could be clinically relevant because a simple blood sample makes possible to individuate a subset of patients (about two third of PD-L1 overexpressing NSCLC population) who may benefit the most from anti-PD-1 therapy (ORR 53%, mPFS 17.2 mo) compared to the remaining one-third of patients having “high” pre-treatment SAA, who contrarily reached a very poor OS (7 months) and whose ORR (7%) and mPFS (2.1 mo) potentially underlie a primary resistance to ICIs, probably including the limited cases of hyper-progression.

The discrimination power of SAA in the selection of patients was not influenced by PD-L1 expression, since the TPS score was not associated with neither SAA level nor response to treatment, and appeared independent from other known prognostic factors, excluding the absence of pleural effusion at baseline which also resulted an independent predictor for better PFS with pembrolizumab, supporting the well-known poor activity of ICIs on pleural metastases. The capability of SAA to predict outcomes of anti-PD-1 treatment was confirmed by the dynamic monitoring of blood protein level, which showed that patients who maintained “low” SAA during therapy reached better PFS and OS than those who maintained “high” SAA or changed the protein level. Furthermore, a subset of patients belonging to “intermediate profile” group change the SAA level from “high” to “low” during the treatment, raising the question whether is the tumor microenvironment favorably modified by immunotherapy increasing likelihood of response during treatment or the variation of the circulating immune-profile primarily influences the activity of ICIs. Early evidence with anti-PD-1 in NSCLC seems support the first hypothesis [29]. Interestingly, a small number of metastatic sites (≤ 2) and thus a low tumor-burden, was the unique clinical characteristic associated with “low” pre-treatment SAA, supporting the fact that blood SAA in cancer patients reflects the direct production by tumor rather than the liver synthesis in response to inflammatory condition. In fact, no relationship was found between SAA high concentration and increased BMI or presence of comorbidities, which were usually characterized by a chronic inflammatory status.

Other systemic inflammation-based prognostic scores have been proposed to improve the selection of patients to candidate to an appropriate therapy or to direct the treatment allocation and the design of randomized clinical trial. In particular, SAA is also included in the acute phase proteins analyzed by the VeriStrat test (Biodesix, USA), a blood-based proteomic assay which assigns patients to either “good” or “poor” status, and was tested as strong independent indicator of prognosis only for tyrosine kinases inhibitors treatment and chemotherapy, while its value for treatment with ICIs remains unknown [30].

Regarding immunotherapy-treated patients, neutrophil lymphocyte ratio (NLR) have been reported repeatedly to have prognostic value, regardless of treatment considered [31]; however, a combined score (the LIPI score) including LDH and derived-NLR, had showed potential predictive value in large retrospective real-life cohort of patients treated with IO [22]. Unfortunately, when applied to randomized patients in clinical trials, the LIPI score revealed as survival predictive biomarker irrespective of treatment administered [23, 32].

These unsuccessful but compelling data underline the great challenge to identify a reliable biomarker with a clear predictive value among the systemic inflammation components, since inflammatory response have a well-recognized prognostic significance in cancer patients across the histotype and treatments employed [33].

Besides the role of SAA, our exploratory study also gave a “real-life” description of efficacy of pembrolizumab prospectively assessed in PD-L1 over-expressing NSCLC patients. The RR (38%) and mPFS (8 mo) observed in our entire cohort were slightly lower compared with those reported in KEYNOTE-024 (ORR 44.8%, mPFS 8 mo) after a similar median time of follow-up [6]. These differences could be explained by the more accurately selected population enrolled in the trial, in which patients with ECOG PS 2, untreated brain metastases and receiving systemic corticosteroids were excluded. Furthermore, the median age of our patients was higher than the trial population one (70.5 versus 64.5 years).

Despite the relevant results and the prospective design which reduces the risk of bias, our study has some flaws. First, the small sample of patients included do not allow drawing any definitive conclusion about the role of this blood biomarker. Second, the value of SAA adopted as cut-off was derived from the analysis of a single-institution study population and thus need to be validated in larger and external series of patients. Third, radiological assessment was performed locally, lowering the strength of the RR and PFS results. Fourth, although our clinical experience suggests that the production of SAA by the tumor would represent a potential novel mechanism of immune-escape through the induction of an immunosuppressive microenvironment, pre-clinical data supporting evidences are not provided in the present study. To avoid the potential bias discussed, a further study (FoRECATT-2) is currently ongoing including an external multi-institutional validation set of patients and in vitro experiments to assess the effect of SAA on immune response. According to the novel approved indication of pembrolizumab, a cohort of patients treated with the combination of chemotherapy and immunotherapy will be also included.

In conclusion, our study provides to clinicians a strong and simple prognostic blood biomarker which may improve the selection of patients who are more likely to benefit from immunotherapy, avoiding the risk of immune-related toxicities for potentially not-responsive patients and also reducing the financial burden for Healthcare System through a more tailored therapeutic approach. Unlike the other available tissue biomarkers, such as PD-L1 and TMB, whose evaluation requires adequate sample achievable only with invasive procedures and is hampered by relevant spatial and temporal heterogeneity, the SAA dosing could represent a “non-invasive” dynamic biomarker which is assessable in every patient during the treatment, reflecting both disease and immune-environmental changes and allowing for an early detection of those cases with acquired resistance to immunotherapy. Finally, our findings if further confirmed, may support the development of novel therapeutic strategies employing anti-SAA e anti-FPR-2 monoclonal antibody which could improve the activity of ICIs by blocking the potential immunosuppressive action of SAA.

### Electronic supplementary material

Below is the link to the electronic supplementary material.

(PDF 544 kb)
