# Supplementary material for: Blood serum amyloid A as potential biomarker of pembrolizumab efficacy for patients affected by advanced non-small cell lung cancer overexpressing PD-L1: results of the exploratory “FoRECATT” study
Source: Cancer Immunol Immunother. 2020 Nov 24;70(6):1583–92. doi: 10.1007/s00262-020-02788-1 (PMC8139913; doi:10.1007/s00262-020-02788-1)
Supplement: Supplementary file 1 — (PDF 544 kb) [file 262_2020_2788_MOESM1_ESM.pdf]

SUPPLEMENTARY MATERIALS

Fig. 1S - Baseline SAA and RR in CT Cohort

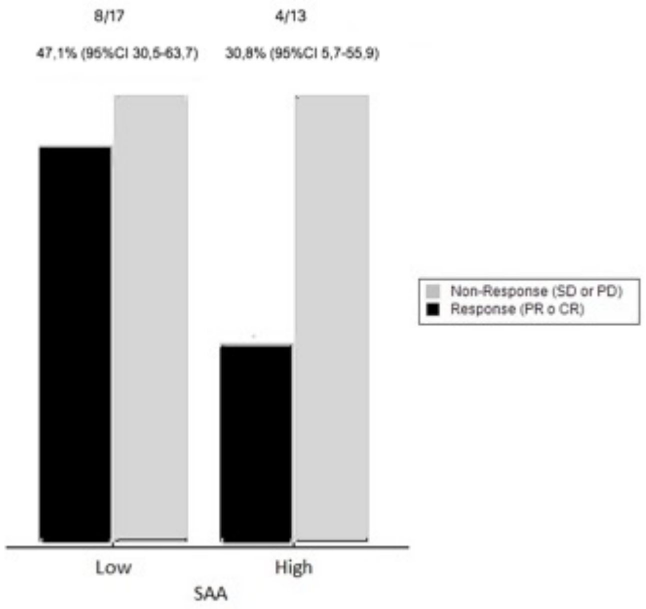

Fig. 2S - PFS (A) and OS (B) according to baseline SAA in CT Cohort

A **B**

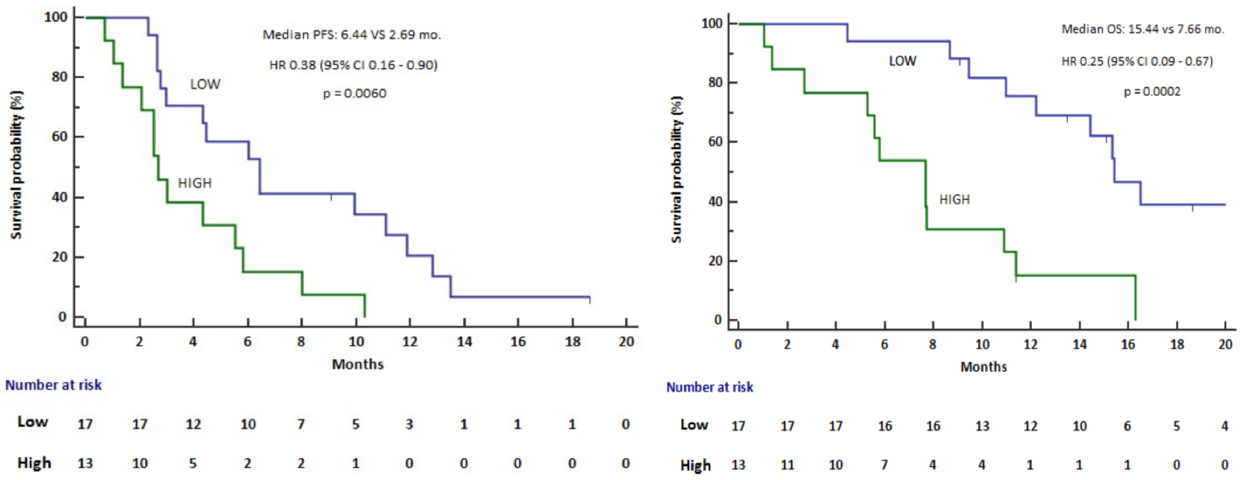

Table 1S – Relationship between Response Rate (RR) and clinical characteristics in P Cohort

		Response		Stable Disease or Progression	
	p value	no	%	n	%
**Age (**in years)	0.330				
<65		4	27	11	73
≥ 65		12	44	15	56
**Sex**	1.000				
Male		11	37	19	73
Female		5	41	7	59
**ECOG PS**	0.790				
0		7	44	9	56
1-2		9	35	17	65
**Smoking history**	1.000				
Never Smokers		2	40	3	60
Current or Ex Smokers		14	38	23	62
**Hystology**	0.380				
Squamous		1	17	5	83
Non Squamous		15	42	21	58
**BMI Categories** (kg/m2)	0.532				
<18.5		1	50	1	50
18.5-24.9		12	43	16	57
≥25		3	25	9	75
**Comorbidities (No vs Yes)**	0.575				
No		9	45	11	55
Yes		7	32	15	68
**No. of Metastatic Sites**	0.209				
1-2		9	50	9	50
≥ 3		7	29	17	71
**Brain Metastases (**No vs Yes)	0.602				
No		9	33	18	67
Yes		7	47	8	63
**Liver Metastases** (No vs Yes)	1.000				
No		15	38	24	62
Yes		1	33	2	67
**Pleural effusion** (No vs Yes)	0.303				
No		13	45	16	55
Yes		3	23	10	77
**Prior thoracic Radioterapy**	1.000				
No		9	38	15	62
Yes		7	39	11	61
**IrAE** (No vs Yes)	0.207				
**No**		11	48	12	52
**Yes**		5	26	14	74
**Steroid Use**	1.000				
No		11	39	17	61
Yes		5	36	9	64

BMI, Body Mass Index; IrAE, Immune-related Adverse Events; no, number

Table 2S - Baseline characteristics of the patients in CT Cohort

	No. Patients (30)	% 100
**Age (years)**		
Median (range)	62.5 (43-83)	-
≥ 65	13	43
<65	17	57
**Sex**		
Male	18	60
Female	12	40
**ECOG Performance Status**		
0	11	37
1	14	47
2	5	16
**Smoking history**		
Current or Former Smokers	18	60
Never Smokers	12	40
**Hystology**		
Squamous	7	23
Non-Squamous	23	77
**No. of Metastatic Sites**		
1	5	17
2	9	30
3	7	23
≥ 4	9	30
**Brain Metastases**		
No	22	73
Yes	8	27
**Site of Mestastases**		
Pleural Effusion	9	30
Liver	3	10
Lung	13	43
Bone	15	50
Mediastinal Nodes	19	63
Adrenal Glands	4	13
Other	4	13
**BMI Categories**		
Underweight <18.5	2	7
Normal weight 18.5-24.9	21	70
Overweight or Obesity ≥25	7	23
**Comorbidities**		
No	3	10
Yes	27	90
**Prior thoracic Radioterapy**		
No	29	97
Yes	1	3
**Steroid Use**		
No	4	13
Yes	26	87

No, number;
